# European Academy of Paediatrics statement on the clinical management of children and adolescents with gender dysphoria

**DOI:** 10.3389/fped.2024.1298884

**Published:** 2024-02-05

**Authors:** Joe Brierley, Vic Larcher, Adamos A. Hadjipanayis, Zachi Grossman

**Affiliations:** ^1^ European Academy of Paediatrics, EAP, Brussels, Belgium; ^2^ Paediatric Bioethics Centre, NIHR Great Ormond Street Hospital Biomedical Research Centre, London, United Kingdom; ^3^ Paediatric Department, Larnaca General Hospital, Larnaca, Cyprus; ^4^ Adelson School of Medicine, Ariel University, Ariel, Israel; ^5^ Maccabi Health Services, Tel Aviv, Israel

**Keywords:** gender dysphoria (GD), ethics, children’s rights, pubertal suppression, transgender (GLBT) issues

## Abstract

Gender issues have become a polarised and political subject in modern paediatrics and indeed, in broader society. These include the management of infants with disorders of sex development and transgender sports participation, but especially recently regarding the management of gender dysphoria. The European Academy of Paediatrics (EAP) acknowledges that there are deeply held beliefs about this issue based on conscience and social norms. Several European countries, led by the UK, have recently reviewed the management of gender dysphoria in children and young people. Recognising the need for far more research into treatments such as pubertal suppression and cross-sex hormones in children and young people, we review the current ethical and legal dilemmas facing children with gender dysphoria, their families and the clinical teams caring for them. We suggest an approach that maintains the child's right to an open future whilst acknowledging that the individual child is the crucial person affected by decisions made and must receive appropriate support in decision-making and care for any associated mental health or psychological issues. Noting that national approaches to this vary and are in flux, the EAP advocates a child-centred individual rights-based analytical approach.

## Introduction

Gender is an essential determinant of personal identity. Typically, gender identity as a boy/man or a girl/woman is congruent with the individual's assigned sex at birth. This century has seen a worldwide increase in both adults and children who do not identify with their biological sex or regard their gender as fluid or non-binary (1). Terminologies describing such individuals have evolved but remain intensely sensitive and emotive; we use transgender to represent all people whose gender identity role or expression differs from the sex registered at birth (1).

People whose gender identity, role, or expression do not conform to social or cultural norms typically associated with their sex assigned at birth are members of the gender-diverse community. Gender nonconformity and diversity is “a common and culturally diverse human phenomenon [that] should not be judged as inherently pathological or negative.” (2) Despite more enlightened social attitudes, some gender-diverse individuals may be subject to discrimination and prejudice. Whilst different models exist (3), the gender minority stress model is important in suggesting that the prejudice and discrimination some individuals endure renders them vulnerable to mental health problems (4, 5). Furthermore, complicated interpersonal and family relationships may produce psychological distress, which is socially induced rather than intrinsic to transgender identity (2).

There is an ongoing, increasingly polarised and vituperative debate about how our current diverse society should treat transgender individuals (especially children) and how their rights should be respected. Increasing numbers of children and adolescents identifying as transgender have led to increased referrals to Gender Identity Development Services (GIDS) or their equivalents, with several European countries, including the U.K., Sweden, Norway, and Finland, having reviewed/are reviewing these services (1, 6–8). Some, consequently, have adopted a more cautious approach to paediatric gender-affirming treatments by restricting some treatments or limiting them to the research environment (4, 6, 9), though none have yet followed some US states in legislating against use in minors (10).

The optimal management of transgender children (both prepubertal and adolescent) remains an area of active controversy and increasingly politicised debate. We highlight the current ethical and legal issues this raises and suggest an approach that accommodates the dynamic nature of gender identity in contemporary society.

## Gender, gender identity development and gender dysphoria (GD) in children

Though children's sense of gender identity typically becomes fixed at 2–4 years of age, atypical gender expressions and behaviours are increasingly recognised as part of normal development and are influenced by broader family and societal factors (11). Typically, they neither persist nor cause distress but occasionally resurface during the questioning of personal identity that can occur during adolescence.

Of note, ICD-11 recently redefined gender identity-related health, replacing ICD-10's “transsexualism” and “gender identity disorder of children” with “gender incongruence of adolescence and adulthood” and “gender incongruence of childhood”, respectively. Both were classified as *Conditions related to sexual health* rather than *Mental and behavioural disorders*, reflecting that trans-related/gender-diverse identity issues are not mental ill-health conditions (12).

Gender differently experienced from biological sex may lead to Gender Dysphoria (GD), defined as a sense of unease because of a mismatch between biological sex and gender identity (13). The severity of discomfort that GD produces in prepubescent children varies and is often transient, with most under GID services not continuing to transition once puberty begins (14). Some younger children with GD exhibit a robust, persistent, and consistent desire to be of the other gender or believe they are of the other gender. They aspire for physical and sexual characteristics to match their experienced gender (15), and can strongly prefer adopting clothing, activities and playmates of their experienced gender, rejecting the physical characteristics, activities and typical expression of their biological gender. However, long-term follow-up studies suggest that over 80% of boys referred for clinical GD to GID services desisted from gender dysphoria in adulthood (16). The role of prepubertal gender social transition in increasing the likelihood of persistence is debated but may provide a means of support (17, 18). Support for transgender children in their expressed gender identity appears to contribute to psychological well-being (19).

Children with severe persisting GD can have severe difficulties as biological secondary sexual characteristics develop. Primary adolescent presentation of GD is a distinct, more severe entity with associated mental health problems, profound distress, and persistent demands for treatment.

## Epidemiological considerations

Data from aggregated statistical surveys indicate that 1.2%–2.7% of children and adolescents identify as transgender (20). GD in prepubescent children is far rarer (0.005%–0.014% birth males; 0.002%–0.003%birth females, DSM-5). Difficulties defining the global prevalence of GD relate to cultural differences in the behavioural expression of gender, their fluctuating, transient nature (21), and cultural acceptability. The recent increases in female GID clinic referrals (1, 15, 22, 23), and the prevalence of Autistic Spectrum Disorders in transgender adults (24), and children (including adolescents) (25), remain unexplained, though some highlight referral pattern changes (26).

One controversial suggestion ascribes the increase in sudden onset GD in adolescence to a social phenomenon termed rapid onset gender dysphoria (ROGD) (27, 28). The argument, initially emerging from interviews with parents of transgender youths, effectively runs that a social contagion fuelled by social media leads to peer group-GD, reflecting a social coping mechanism for other issues. The polarisation of the subsequent debate will be familiar to all, with many experts and scientific bodies critical of the research and concept (26). However, others recognise the need to thoroughly investigate one of the few offered explanations for the recent demographic changes (29).

Detransition, the process of discontinuing or reversing gender transition, can occur in adolescents and young adults, often connected with a change in how the individual identifies or conceptualises their sex or gender since initiating transition. It is complex, the debate similarly polarised, and crucially, detransition should be isolated from the harmful concept of forced conversion. The EAP wholeheartedly agrees with those calling for further understanding, not controversy (30), which represents our position on most of the issues involved in gender dysphoria.

## Harms associated with GD

Transgender children may suffer stigmatisation and discrimination, leading to “minority stress,” (4) and vulnerability to mental health problems. All forms of abuse, including bullying, are more common, (31, 32) as are depression, anxiety and suicidal ideation in children, adolescents, and young adults. (33, 34) Lack of family and social support can lead to social isolation and feelings of rejection, with profound psychosocial consequences, but may be alleviated by appropriate support.

Adolescents with GD are more likely to present with related mental health problems and underlying psychopathology compared with cisgender individuals (35). Those implacably convinced of their gender identity may be at risk from unregulated internet access to unsafe drugs. Despite legislation in several European countries, so-called conversion treatment remains lawful in some and actively practised by some communities. The EAP is clear that treatment to convert transgender children to stereotypically gendered individuals against their will fails to respect their identity, compounds distress and is unethical (36, 37).

## Current treatment protocols for children and young people with GD

All treatments should help children feel comfortable in their gender identity and support them in facing issues that arise (38). Many current protocols follow a staged approach (the Dutch protocol), with progressively more invasive, less reversible interventions (22, 39). Comprehensive multidisciplinary clinical and psychosocial assessment of both child and family, with counselling and support, precedes the following sequence: (1) Suppression of puberty by Gonadotropin-Releasing Hormone analogues (GnRH-a: Puberty Blockers); (2) Administration of gender-affirming cross-sex hormones; (3) Gender-affirming surgery. Completion of one stage is usually necessary, but not invariable, before continuation to the next, and some guidance supports surgery without hormone therapy (2, 40). In Europe, stage 2 is usually deferred until the legal age of consent (often 16 years but variable see below) and stage 3 until adulthood. Although the process can be halted at any stage, concerns about the practical difficulties of doing so and doubts about long-term outcomes have led to international reconsideration of this approach.

Ideally, the initial assessment should involve confirmation of diagnosis (including severity, duration and impact), exploration of the child's views, preferences, hopes, and expectations, and broad psychosocial assessment, including the opinions of the family and relevant others, as such it helps determine the child's best interests. Psychosocial support for the child and family is mandatory. Social transition, where the child experiences life in their chosen gender without medical intervention, may be tried.

The original Dutch protocol had strict entry criteria, including age >12, persistent and severe dysphoria, worsening at puberty, an absence of other psychopathology and family support. Over time, some of the elements of the protocol appear to have been extended to include younger people, including prepubescent children, outside clinical trials. This gender-affirming approach has been both criticised and supported (40, 41). Most controversy has centred on the use of GnRH-a to block puberty in peri-pubescent children (especially given the earlier onset of puberty seen in children today) due to potential long-term health and fertility consequences and younger children's lack of capacity to consent (23).

## Fertility preservation in children with GD

Treatments that delay endogenous puberty may impact later fertility. Children may be reluctant to stop puberty-suppressing agents, and once stopped, gamete production can be slow to resume. It is important to discuss fertility risks and fertility preservation options with transgender individuals and their families prior to initiating treatments that may compromise future reproductive function (2, 39). Despite routine counselling, few GD youths opt for gamete harvest (42).

EAP, as a paediatric society, supports children and young people's decision-making about their health in line with the UNCRC norms and national legislation. However, our arguments will develop into advocating for a rights-based individualised analytical approach for children and young people, which should maintain an open future for them. Therefore, we must give significant consideration to approaches that maintain future fertility.

## Gonadotropin-releasing hormone analogues (GnRH-a) in children with GD

GnRH-a inhibit puberty by action on gonad development, suppressing the external appearance of puberty. Although widely licensed to treat precocious puberty, use inGD is controversial, not least whether use is experimental or innovative (43, 44). Some countries now restrict GnRH-a use to research (45), or to an exceptional/case-by-case basis (45).

The rationale for GnRH-a is to permit ongoing assessment, reflection, support, and counselling free from the anxieties and stress arising from puberty in an undesired gender. Suppressing unwanted secondary sexual characteristics may reduce the extent of any later gender-affirming surgery to complete transition. An ancillary purpose is the reduction of anxiety and depression with increased well-being. The efficacy of GnRH-a in achieving any of these is debated (23), but crucial to the harm/benefit analysis required for each child. This analysis is particularly challenging as some potential harms, e.g., growth suppression and reduced bone density, are well-recognised, but others less so. The potentially harmful bio-psychosocial consequences of delaying puberty must be balanced against the amelioration of GD-induced distress, with efficacy reported in one uncontrolled study (46), but methodological concerns about other studies means their use remains controversial (23). GnRH-a use may irrevocably lead to the use of trans-sex hormones and surgical transition (47, 48), so it may arguably compromise rather than facilitate freedom of choice.

Several of the national European reviews concluded that the few limited quality studies on puberty blockers in GD, mental health, and quality of life provide a very low certainty of efficacy (49, 50). The recognised ethical and practical difficulties of performing controlled trials do not preclude the need for either appropriate comparator studies or long-term follow-up research (1, 31).

The fundamental question of whether biomedical treatments (including hormone therapy) for gender dysphoria are effective remains contested. Although de Vries' original study was persuasive, others have questioned efficacy, and as Clayton highlights, “there is no robust empirical evidence that puberty blockers reduce suicidality or suicide rates.” (51)

Nevertheless, even if GnRH-a treatment is considered experimental, it may be ethically justifiable on compassionate grounds, subject to specific agreed-upon and validated criteria and independent peer review (52).

## Gender-affirming transgender hormone therapy

Sex hormones, widely used in adult medicine, are rarely used in paediatrics. Transgender hormones are rarely used in Europe before the age at which children can consent without parental knowledge, reflecting unease at the limited evidence of efficacy but considerable long-term consequences (2, 39, 53). Some information is derived from the standard use of such agents to cause an earlier end to puberty, thereby restricting the final height of some tall adolescents. A more controversial use outside Europe is to restrict the growth of children with severe neuro-disability (54).

## Surgery

In Europe, unlike the US, both radical and more cosmetic transgender surgery seems confined mainly to adults with capacity (55). The exception seems to be mastectomies practised in the Netherlands and France from age 16 on a case-by-case basis (50). Surgery for trans-men can involve (i) gender surgery, including construction or implant of a penis and scrotum and hysterectomy and salpingo-oophorectomy, and (ii) bilateral mastectomy and chest reconstruction with nipple repositioning, dermal implant and tattoo. Surgery for trans-women can include (i) gender surgery with orchidectomy and penectomy, with vaginoplasty, vulvoplasty and clitoroplasty and (ii) chest surgery with breast implants. Furthermore, for trans-women who have progressed through male puberty, throat surgery to reconstruct the larynx can occur, as well as facial feminisation surgery and hair transplants. Discourse about whether such surgery should be funded by struggling public health systems or is the right of individual transgender citizens is as polarised and country-dependent as other transgender issues.

Given that later arguments will support the child's right to an open future, our view as paediatricians is that it is correct to defer irreversible surgery until adulthood.

## Ethical and legal considerations of GnRH-a use and transgender hormones in children

The use of these drugs in children with GD raises tensions between ethics and law about who should determine a young person's best interests and provide consent.

Application of ethical principles and appeal to children's rights may justify use in the best interests of transgender children. Further justifications include respect for the child's personal identity and evolving autonomy. Puberty blockers may be justified in those severely troubled by lack of congruence between gender identity and sex, as delaying puberty and proceeding to transition may be less harmful than the long-term consequences of GnRH-a therapy. However, the lack of long-term outcome data, including the psychosocial consequences of delayed puberty, is troubling and should be the focus of further research before further expansion of their use.

The law may play a role in determining whether GnRH-a and gender-affirming treatments are in a child's best interests but does not usually address who is best placed to determine them. Courts examine the relevant facts and the beliefs and values of the parties in any particular case to determine best interests.

Because decisions are intensely personal and go to the core of personal identity, some argue that authority should rest with the young person/adolescent (from puberty to 18 years) as decisions impact them most, irrespective of their capacity, provided they can exercise minimal autonomy (24). Court involvement may compound a child's distress and delay access to treatment.

Most European countries use a clinically determined gender-affirming process rather than a self-decided process (56). Despite European bodies' calls to affirm transgender individuals' right to freedom from discrimination and to healthcare (57), many European countries restrict access to treatment (58). As highlighted, many member states restrict puberty blockers in children. To date, 18 US states have outlawed all gender-affirming treatments, including puberty blockers, in those under 18 (59). In Australia, gender-affirming treatment can only be given to those under 18in the absence of dispute between relevant parties about the young person's capacity, diagnosis of gender dysphoria or treatment proposed (60).

## Consent for puberty blockers, transgender hormones and gender surgery in children

The age of presumed consent varies between European jurisdictions (61) and does not align with access to transgender hormone therapy (62), and the situation with access to puberty blockers is fast changing. Whether parental consent supporting their child's preference is adequate is also unclear, changing and varies between countries. However, the positive role of parental, social and peer support in decision-making is recognised.

The appropriate mechanism for obtaining consent for puberty blockade and gender-affirming therapies in GD is disputed. Such treatments go to the core of personal identity and autonomy, with far-reaching implications for both child and family. Excluding parents from providing consent maximises respect for the young person's autonomy and personal identity but is controversial, given that 59% of paediatric GID-service referrals are under 16 (1).

Children's competence to make such complex decisions has been questioned because of a lack of life experience and the immaturity of executive decision-making (63). Furthermore, the distress and anguish caused by gender incongruity may produce a situational incapacity where a child cannot assimilate and use information to make the decision at hand. No matter how much it is orientated to the child's understanding, providing more information may not materially alter the situation.

Some, therefore, believe children lack the capacity to make decisions that profoundly impact their future lives, relationships, and reproductive abilities. Others regard the matter of capacity as less important than respecting the unique lived experience of trans-youth in supporting their decision-making authority, especially if supported by parents (24). This approach seems plausible given that some under 16-year-olds have been allowed, with parental support, to reject life-prolonging treatments such as heart transplantation, whereas others provide valid consent to harvesting/storing of gametes facing cancer treatment-induced infertility.

Questions remain as to who may request or consent to puberty blockers or transgender hormones in children with GD, whether legal intervention or even proscription is ethical or practical, and what instruments may help address this.

Some centres have specifically explored medical decision-making competence in transgender youth (64). Our view is that such decision-making competence is essentially part of daily paediatric practice with young people participating in experimental cancer therapies, discussing future fertility and gamete preservation, mental health treatments and high-risk transplant surgery. Constrained by national law, we consider the ideal decision-making to be a child-centred combination of the fully informed child/young person's emerging autonomy, the expert clinical team informed by up-to-date literature and consensus/second opinions and the child's parents/those with legal decision-making capacity for the child.

## Broader ethical perspectives in the treatment of gender dysphoria and nonconformity in children

In the following sections, we choose to use the term child to refer to an individual under 18 years of age. Relieving gender dysphoria is consistent with health professionals' universally recognised duty of care and “treating children and young people as individuals and respecting their views, as well as considering their physical and emotional welfare.” (65) Maximising the welfare of children is consistent with the guiding principle that doctors should always act in the best interests of children and young people in all decisions affecting them and is inherent in the articles of the UN Convention on the Rights of the Child. (66)

In determining best interests, the expressed views of the child or young person, including consistency over time, should be considered together with the balance of potential benefits and harms of proposed interventions (GMC18). The decision-making authority of parents and families may be reduced in decisions relating to the treatment of transgender youth due to the intensely personal nature of the decision and its significant impact on the child's identity. Nevertheless, the role of social media, support groups and peer pressure, as well as that of others close to the child, should also be considered. Indeed, the role of social media in not just ROGD but more broadly in GD and perhaps separately in the increase in childhood mental health problems is overdue serious academic exploration (67). It is also worth noting that the specific views of any professional team are also likely to influence treatment options. So, teams treating GD should conform to appropriate professional standards and operate within clear governance structures with regular peer review.

Applying libertarian principles, including that of subsidiarity (68), would support a permissive gender-affirmative approach. However, EAP supports a more balanced approach, focussed on interventions that least restrict the transgender child's future options whilst trying to prevent significant or serious harm.

Balancing these factors depends on the child's particular circumstances, so unjustified assumptions about best interests must be avoided. Consideration of intersectionality is vital given that other factors such as sexuality, race and disability may influence the presentation. Clinicians must address specific mental health needs in a child with GD, such as an autistic spectrum disorder.

## A rights-based analytical approach

Current protocols for treating transgender children reinforce a biologically determined binary concept of gender, with which we disagree. Such an approach seems counter to current social constructs of gender identity that are more complex, diverse, and fluid than defined by biological constraints. The changing interplay of factors that shape the child's identity as they mature needs to be acknowledged within the framework of children's rights. Treatment protocols should genuinely reflect this complex and evolving landscape.

A rights-based analytical approach may provide sufficient agility to accommodate this dynamic situation and support the child's transition and exploration. It can recognise and support agency, participation and empowerment and help acknowledge and address the competing rights of relevant parties while retaining focus on the child in question.

The fundamental premise is that the child's best interests are a primary consideration (Article 3 UNCRC) (43), an approach consistent with a detailed clinical and psychosocial assessment. Application of protection rights supports therapeutic interventions that protect the child from discrimination (Article 2 ibid), provide the highest attainable standards of care, and deliver necessary access to it (Article 24 ibid). Liberty rights entail that children capable of forming views should have the freedom to express them, and their views accorded due weight in accordance with their age and maturity (Article 12 ibid). A rights-based approach also acknowledges the importance of family and broader social support.

Problems remain in deciding whether children can plausibly claim liberty rights, especially in a request for invasive, non-reversible treatment, where the child’s right to protection from the harms of invasive, irreversible gender-affirming treatment must be carefully considered.

A further consideration is the child's right to an open future, which protects the child against having life choices made for them until they choose for themselves (69). A child entering puberty in a gender they do not identify with has their right to an open future compromised, but a child on a path to transition they may later regret is equally compromised. An alternative approach that better retains options and permits more fluidity of gender expression and identity seems preferable despite its essential ambiguities and difficulties in balancing competing rights. Constructively and openly addressing these issues could improve the decision-making process.

## Future considerations

Approaches to questions of gender identity, provision of puberty blockers and gender-affirming treatments in European children are currently under critical review. Fresh guidance will likely be issued, though specific legislation seems unlikely. Traditional medico-legal approaches to GD have been increasingly scrutinised. The role of parental support and decision-making authority in the consenting process needs further exploration. Social media platforms will continue to provide a forum both for those who support a more liberal approach to transgender issues and those who believe such an approach is fundamentally flawed.

We argue that a nuanced, non-binary approach is consistent with respect for liberty, protection rights, and the best interests principle. It provides the possibility of an open future that includes future relationships and fertility preservation, allows parents to support the consent process, and protects those for whom this is not possible. Finally, it avoids a dichotomised deterministic yes/no Male/Female approach to children and young people's sense of gender identity by allowing gender fluidity. EAP considers this approach preferable and suggests we must move away from the polarised confrontational approach that has mired rational debate.

## Conclusions and recommendations from EAP

### Conclusions

The treatment of transgender children raises important questions concerning personal identity and autonomy. Treatment protocols, their clinical, ethical and legal foundations, who should determine them, and how they should be applied are controversial and will continue to produce polarised opinions. The balance between respecting a young person's developing autonomy and protecting them from harm remains crucial. We suggest that a flexible, consensus-building, rights-based approach, supported by a robust understanding of the relationship between biological sex and gender, is in children's best interests and supports their right to an open future.

An international research programme to define optimal treatment and outcomes, based on meticulous observation and comparator studies, should be urgently funded and performed. In the interim, children and parents must receive appropriate support and care while issues are resolved.

### Recommendations from EAP

• EAP recognises that in different countries, there will be a variety of approaches to this complex issue.
• EAP recommends an individual rights-based analytical approach to caring for young people with gender dysphoria.
• As this is a rare condition, referral to fully funded expert paediatric centres is necessary to develop the specialist services we recognise deliver the optimal care for young people and their families.
• EAP urges urgent research into the optimal approach to supporting young people with gender dysphoria and their families.
• Paediatricians or other physicians (i.e., GP/family doctor) who care for children and adolescents should support those with gender dysphoria, which includes directing them to a multidisciplinary team of experts and providing ongoing primary and tertiary care support.
• Thus, paediatricians and all healthcare professionals treating children and adolescents should be well-trained on gender issues.
• EAP suggests there should be further understanding, not controversy, in gender dysphoria.

## Data Availability

The original contributions presented in the study are included in the article/Supplementary Material, further inquiries can be directed to the corresponding authors.
